# The contribution of psychological distress to socio-economic differences in cause-specific mortality: a population-based follow-up of 28 years

**DOI:** 10.1186/1471-2458-11-138

**Published:** 2011-02-28

**Authors:** Kirsi M Talala, Taina M Huurre, Tiina KM Laatikainen, Tuija P Martelin, Aini I Ostamo, Ritva S Prättälä

**Affiliations:** 1 Department of Health, Functional Capacity and Welfare, National Institute for Health and Welfare (THL), Helsinki, Finland; 2 Department of Mental Health and Substance Abuse Services, National Institute for Health and Welfare (THL), Helsinki, Finland; 3 Department of Chronic Disease Prevention, National Institute for Health and Welfare (THL), Helsinki, Finland; 4 Thematic Programmes Unit, National Institute for Health and Welfare (THL), Helsinki, Finland; 5 Tampere School of Public Health, University of Tampere, Tampere, Finland

## Abstract

**Background:**

Psychological factors associated with low social status have been proposed as one possible explanation for the socio-economic gradient in health. The aim of this study is to explore whether different indicators of psychological distress contribute to socio-economic differences in cause-specific mortality.

**Methods:**

The data source is a nationally representative, repeated cross-sectional survey, "Health Behaviour and Health among the Finnish Adult Population" (AVTK). The survey results were linked with socio-economic register data from Statistics Finland (from the years 1979-2002) and mortality follow-up data up to 2006 from the Finnish National Cause of Death Register. The data included 32451 men and 35420 women (response rate 73.5%). Self-reported measures of depression, insomnia and stress were used as indicators of psychological distress. Socio-economic factors included education, employment status and household income. Mortality data consisted of unnatural causes of death (suicide, accidents and violence, and alcohol-related mortality) and coronary heart disease (CHD) mortality. Adjusted hazard ratios were calculated using the Cox regression model.

**Results:**

In unnatural mortality, psychological distress accounted for some of the employment status (11-31%) and income level (4-16%) differences among both men and women, and for the differences related to the educational level (5-12%) among men; the educational level was associated statistically significantly with unnatural mortality only among men. Psychological distress had minor or no contribution to socio-economic differences in CHD mortality.

**Conclusions:**

Psychological distress partly accounted for socio-economic disparities in unnatural mortality. Further studies are needed to explore the role and mechanisms of psychological distress associated with socio-economic differences in cause-specific mortality.

## Background

Socio-economic inequalities in mortality are well reported in Western European countries [1-5]. Excess coronary heart disease mortality [6,7] as well as unnatural mortality, namely suicide [8-10], alcohol-related deaths [9,11,12], and accidental and violent causes of death [13], have been reported in lower socio-economic groups. Socio-economic variation is also significant in Finland in these specific causes of death [10,11,14,15]. Over the past 40 years, the socio-economic inequalities in mortality have widened in several countries [16,17].

In their search for new explanations for socio-economic disparities, scholars exploring the links between socio-economic position and health are moving beyond the material and behavioural factors, which do not fully account for these disparities. Psychological indicators, such as negative emotions (including depression and anxiety) [18], stress [19,20] and insomnia [21], have been proposed as a plausible explanation for the socio-economic gradient in health. It is suggested that socio-economic differences in health are at least partly mediated by psychological distress stemming from socio-economic deprivation. Supposedly, not only absolute deprivation, but also relative deprivation, that is, one's position in the hierarchy vis-à-vis others, is important and associated with health [22].

In Williams' [23] conceptual framework, psychosocial factors (consisting of risky health practices, social ties, perception of control, stress and affective states) are seen as critical mediators between social structure and health status. Marmot and Brunner [22] proposed a model in which social structure is linked to health and disease via material, psychosocial and behavioural pathways. These approaches view psychological distress not as the property of an individual but as the response of the individual to the external environment acting upon him or her. According to Schnittker [24], the resources provided by socio-economic position are related to the inferences individuals draw about the self, and these psychological states might affect physical and mental health. Wilkinson states that the psychological pain resulting from low social status affects patterns of violence, disrespect, shame, poor social relations and depression [25].

An association between lower socio-economic position and poor mental health [26-30] has been reported, as well as an excess mortality associated with psychological distress, especially with death from unnatural causes and cardiovascular disease [31-37]. The evidence regarding the potential mediating role of psychological factors on the relationship between socio-economic position and health is not unambiguous. Schnittker [24] examined whether four psychological factors (self-esteem, mastery, neuroticism and depressive symptoms) mediated the relationship between socio-economic position and three indicators of health (self-rated health, functional limitations and chronic conditions) and found only weak mediating effects. The results provided the strongest evidence for mediation in cases of neuroticism or depressive symptoms. Marmot et al. [38] found evidence for the mediation of psychological well-being measures (control/self-efficacy) on the association between education and health. Likewise, a Hungarian population study found that depressive symptom severity mediated between relative socio-economic deprivation and higher self-rated morbidity rates [36].

To our knowledge, few studies have examined the question of whether psychological factors contribute to socio-economic differences in cause-specific mortality. It has been proposed that psychological factors play a mediating role in the socio-economic differences associated with cardiovascular mortality [7,39]. In a U.S. study [40], psychological distress as measured by hopelessness, depression and life dissatisfaction was not a significant contributor to socio-economic disparities in all-cause mortality. Van Oort et al. [41] found that, when independent of material factors, psychosocial factors contribute little to the explanation of educational inequalities in all-cause mortality.

### Aim of the study

The aim of this study was to explore whether self-reported psychological distress, measured by depression, stress and insomnia, mediates socio-economic differences (indicated by educational level, employment status and household income) in unnatural (suicide, accidents and violence, and alcohol-related mortality) and coronary heart disease (CHD) mortality in the 28-year follow-up (see Additional file 1). In other words, the aim of the study is to examine the contribution of psychological distress to relative differences in cause-specific mortality by socio-economic position.

## Methods

### Data

The basic data source is the nationwide, repeated cross-sectional survey, "Health Behaviour and Health among the Finnish Adult Population", conducted annually since 1978 by the National Public Health Institute of Finland [42]. The survey questionnaire is mailed to a random sample of 5,000 Finns aged 15-64 years. The simple random sample was conducted by The Finnish Population Information System which is a computerized national register that contains basic on-line information about all Finnish citizens residing permanently in Finland. The survey years covered in this study are 1979-2002. The year 1985 has been excluded from the survey due to missing personal identification codes for that year. Respondents under 25 years of age have been excluded from this study because their socio-economic status is not established.

We have supplemented the survey data with education and household income variables from Statistics Finland Register Data from the years 1979-2002 and the Finnish National Causes of Death Register follow-up data from the years 1979-2006. The mortality data include immediate, contributing and underlying causes of death, as well as the exact date of death. The data linkages were derived by using the personal identification codes assigned to all persons living permanently in Finland. After excluding the missing data on psychological distress variables (N = 1129, 1,6%), the total number of cases was 67871 (average annual response rate 73.5%), out of which 32451 were men (average response rate 69%) and 35420 were women (average response rate 78%). Our study is reviewed and supported by The Institutional Review Board of National Institute for Health and Welfare, (THL) (IRB 00007085, FWA 00014588).

### Psychological distress variables

We questioned the respondents about 14 health problems or symptoms, among them depression and insomnia, by the following single question: "Have you had any of the following symptoms or health problems during past 30 days?" (Yes, if so). Stress was addressed in a separate question on a four-point scale (1 = unbearable situation, 4 = no stress at all); respondents were asked if they had symptoms of tension or had been under great stress or considerable strain during the past 30 days. We considered unbearable stress as having the most negative effect on health and mortality and being associated with social disadvantage. Therefore, we classified those reporting an unbearable situation as having stress. We investigated the correlation of the psychological distress measures in another paper using this same data and showed that single-question depression (males r = .58; females r = .55) and insomnia (males and females r = .38) correlated with the general mental health inventory (MHI-5) [30]. In this study, self-reported psychological distress is thought to reflect the subjective experience of psychological well-being, and it is used to explore the role of psychological distress in generating socio-economic differences in mortality at an extensive population level [43].

### Socio-economic variables

Socio-economic variables included education and household income from the register data and employment status from the survey questionnaire. We collected the register data for education and income from 1980 statistics for the survey years 1979-1983, from 1985 statistics for the survey years 1984-1986 and annually from 1987 until 2000. For the survey years 2001-2002, we collected the socio-economic data from the year 2000.

The educational level was derived from the Register of Educational Qualifications and Degrees, which follows, as far as possible, the principles and categories of the revised UNESCO International Standard Classification of Education 1997 (ISCED 1997). We divided the respondent's educational qualification into three categories: the lowest level included respondents with no education, an unknown education or with lower secondary education; the intermediate level included respondents with upper secondary or post-secondary non-tertiary education; and the highest level included respondents with tertiary education.

Household income has been found to be more strongly and consistently associated with health than individual income. [44] We calculated household income as taxable total gross income for a household per year without transfer payment, divided by the consumption unit of the OECD equivalence scale. The first adult in the household was weighted as 1.0, other adults as 0.7 and children under 18 as 0.5 [45]. We further divided household income per consumption unit into tertiles by every study year, in order to keep the comparability of the variable over time.

Employment status during most of the year consisted of the categories of employed and unemployed. Additional categories, that is housewives/husbands, students and retired people were excluded from the analyses concerning mortality differences by employment status.

### Mortality

In this study we analysed unnatural causes of death like suicide, accidents and violence, and alcohol-related mortality, and, for purposes of comparison, a general cause of death, coronary heart disease (CHD) mortality. We identified the causes of death using the International Classification of Diseases (ICD, WHO; 1974, 1978, 1992). The 8th revision was used for the years 1979-1986, the 9th revision for the years 1987-1995 and the 10th revision for the years subsequent to 1996. The classifications for suicide were E950-E959 (ICD-8 and ICD-9) and X60-X84 (ICD-10). Accidents and violence were ICD codes E800-E859, E861-E949, E960-E999 (ICD-8), E800-E849, E852-E949, E960-E999 (ICD-9) and V00-V99, W00-W99, X00-X44, X46-X59, X85-X99, Y00-Y89 (ICD-10). The definition of alcohol-related deaths included injuries, diseases and poisonings where alcohol was the main cause of the death (ICD-8 codes 291, 303, 571.0, 577, and E860; ICD-9 codes 291, 303, 357.5, 425.5, 535.3, 571.0-571.3, 577.0D-577.0F, 577.1C-577.1D and E851; ICD-10 codes F10, G31.2, G62.1, G72.1, I42.6, K29.2, K70, K86.0, O35.4 and X45). We grouped suicides, accidents and violence and alcohol-related deaths together and called them 'unnatural' causes of death. The classification for coronary heart disease (CHD) mortality was 410-414 for ICD-8 and 9 codes and I20-I25 for ICD-10 codes.

### Statistical methods

For preliminary analyses, we examined associations between psychological distress and socio-economic position with a logistic regression model reporting odds ratios (OR) with 95% confidence intervals (CI) (see Additional file 2) and between psychological distress and mortality with a Cox proportional hazard model reporting hazard ratios (HR) with 95% confidence intervals (CI) (see Additional file 3) [46].

We conducted the main analyses using the Cox proportional hazard model (Tables 1, 2, 3). All the analyses were performed with age and study year as covariates. Variation over time was taken into account by adjusting for the study year. To take into account the non-linear association of age with unnatural mortality, we adjusted mortality analyses for age squared. We carried out all the statistical analyses separately for men and women, using the statistical package SPSS 17.0 for Windows (SPSS Corporation 2008).

**Table 1:** The effect of adjusting for self-reported psychological distress on educational level differences in excess mortality.

	Unnatural mortality					CHD mortality				
Educational level	High	Intermediate		Low		High	Intermediate		Low	
Men		HR (95% CI)	(%)	HR (95% CI)	(%)		HR (95% CI)	(%)	HR (95% CI)	(%)
1: confounders*	1	1.43 (1.15-1.77)		1.58 (1.28-1.94)		1	1.19 (0.98-1.43)		1.36 (1.16-1.59)	
1+depression	1	1.40 (1.13-1.74)	-7	1.53 (1.25-1.89)	-9	1	1.18 (0.98-1.43)	-5	1.36 (1.16-1.59)	0
1+stress	1	1.41 (1.14-1.75)	-5	1.55 (1.26-1.91)	-5	1	1.18 (0.98-1.43)	-5	1.36 (1.16-1.59)	0
1+insomnia	1	1.41 (1.13-1.74)	-5	1.53 (1.24-1.88)	-9	1	1.19 (0.98-1.43)	0	1.36 (1.16-1.59)	0
1+all psychological distress variables	1	1.38 (1.11-1.72)	-11	1.51 (1.23-1.85)	-12	1	1.18 (0.98-1.43)	-5	1.35 (1.15-1.58)	-3
Women										
1: confounders*	1	1.29 (0.88-1.89)		1.16 (0.79-1.69)		1	1.54 (1.02-2.31)		2.32 (1.62-3.31)	
1+depression	1	1.28 (0.87-1.87)	-3	1.13 (0.77-1.65)	-19	1	1.52 (1.01-2.28)	-4	2.27 (1.59-3.24)	-4
1+stress	1	1.28 (0.88-1.88)	0	1.13 (0.77-1.65)	-19	1	1.53 (1.02-2.29)	-2	2.29 (1.60-3.27)	-3
1+insomnia	1	1.29 (0.88-1.90)	0	1.14 (0.78-1.67)	-19	1	1.53 (1.02-2.30)	-2	2.30 (1.61-3.28)	-2
1+all psychological distress variables	1	1.27 (0.87-1.86)	-7	1.11 (0.76-1.62)	-31	1	1.51 (1.01-2.27)	-6	2.25 (1.57-3.21)	-5

Hazard ratios (95% CIs) and percent reduction (%) in mortality among those with an intermediate or low education compared to those with a high education after adjusting for psychological distress. *The confounders: age, age squared, study year.

**Table 2:** The effect of adjusting for self-reported psychological distress on employment status differences in excess mortality.

	Unnatural mortality			CHD mortality		
Employment status	Employed	Unemployed		Employed	Unemployed	
Men		HR (95% CI)	(%)		HR (95% CI)	(%)
1: confounders*	1	4.12 (3.26-5.21)		1	2.06 (1.58-2.68)	
1+depression	1	3.50 (2.75-4.44)	-20	1	2.00 (1.53-2.59)	-6
1+stress	1	3.78 (2.98-4.79)	-11	1	2.03 (1.56-2.64)	-3
1+insomnia	1	3.45 (2.72-4.36)	-21	1	2.01 (1.55-2.62)	-5
1+all psychological distress variables	1	3.15 (2.47-4.00)	-31	1	1.96 (1.50-2.56)	-9
Women						
1: confounders*	1	3.50 (2.13-5.75)		1	1.91 (1.18-3.11)	
1+depression	1	3.09 (1.87-5.11)	-16	1	1.83 (1.12-3.00)	-9
1+stress	1	3.22 (1.95-5.32)	-11	1	1.91 (1.18-3.11)	0
1+insomnia	1	3.15 (1.91-5.19)	-14	1	1.88 (1.16-3.07)	-3
1+all psychological distress variables	1	2.86 (1.73-4.73)	-26	1	1.83 (1.12-2.99)	-9

Hazard ratios (95% CIs) and percent reduction (%) in mortality among the unemployed compared to the employed after adjusting for self-reported psychological distress. *The confounders: age, age squared, study year

**Table 3:** The effect of adjusting for self-reported psychological distress on household income differences in excess mortality.

	Unnatural mortality					CHD mortality				
Income	High	Intermediate	(%)	Low	(%)	High	Intermediate	(%)	Low	(%)
Men		HR (95% CI)		HR (95% CI)			HR (95% CI)		HR (95% CI)	
1: confounders*	1	1.19 (0.98-1.44)		1.70 (1.42-2.04)		1	1.37 (1.19-1.58)		1.58 (1.38-1.81)	
1+depression	1	1.16 (0.95-1.40)	-16	1.62 (1.35-1.94)	-11	1	1.36 (1.18-1.57)	-3	1.56 (1.36-1.79)	-3
1+stress	1	1.18 (0.97-1.43)	-5	1.65 (1.38-1.98)	-7	1	1.37 (1.19-1.58)	0	1.57 (1.38-1.80)	-2
1+insomnia	1	1.19 (0.98-1.45)	0	1.65 (1.38-1.98)	-7	1	1.37 (1.19-1.58)	0	1.57 (1.38-1.80)	-2
1+all psychological distress variables	1	1.18 (0.97-1.43)	-5	1.59 (1.33-1.92)	-16	1	1.37 (1.19-1.57)	0	1.56 (1.36-1.78)	-3
Women										
1: confounders*	1	1.08 (0.75-1.55)		1.80 (1.29-2.50)		1	1.37 (1.07-1.75)		2.13 (1.71-2.65)	
1+depression	1	1.07 (0.75-1.54)	-13	1.73 (1.24-2.40)	-9	1	1.36 (1.06-1.74)	-3	2.09 (1.68-2.60)	-4
1+stress	1	1.08 (0.75-1.54)	0	1.73 (1.24-2.40)	-9	1	1.37 (1.07-1.75)	0	2.10 (1.69-2.62)	-3
1+insomnia	1	1.09 (0.76-1.56)	+13	1.77 (1.27-2.46)	-4	1	1.37 (1.07-1.75)	0	2.11 (1.69-2.63)	-2
1+all psychological distress variables	1	1.08 (0.75-1.55)	0	1.69 (1.21-2.35)	-14	1	1.36 (1.06-1.74)	-3	2.07 (1.66-2.58)	-5

Hazard ratios (95% CIs) and percent of reduction (%) in mortality among intermediate or low household income levels compared to high income level after adjustments of self-reported psychological distress.*The confounders: age, age squared, study year

In the main Cox proportional hazard analyses, we first carried out the base model to explore the relative differences in mortality outcomes by socio-economic variables adjusted for age, age squared and study year. In the following models, we adjusted for each of the psychological distress variables separately and, finally, for all of them simultaneously to see whether those variables contributed to the socio-economic disparities in mortality. To assess the impact of the adjustment of different variables on the base model hazard ratio, we calculated the percentage reduction of the HR as follows: [(base model HR-base model plus other factors HR)/(base model HR-1)] × 100 [7,47]. We interpreted the reduction in the hazard ratio to tell how much of the association between the individual socio-economic variables and mortality was accounted for by the measures of psychological distress.

## Results

Table 4 describes the follow-up data by socio-economic position. The total number of deaths for unnatural causes was 716 for men and 222 for women, while the numbers for CHD mortality were 1,389 for men and 635 for women. Fourteen per cent of men and 18% of women reported depression, 18% of men and 19% of women reported insomnia, and 2.6% of men and 2.4% of women reported unbearable stress (not shown in the table).

**Table 4:** Description of the mortality follow-up data by socio-economic position among men and women

	Men					Women				
	**N**	**Person years at follow- up**	**N deaths (unnatural mortality)**	**N deaths (CHD mortality)**	**Mean follow-up time (years)**	**N**	**Person years at follow- up**	**N deaths (unnatural mortality)**	**N deaths (CHD mortality)**	**Mean follow-up time (years)**

Education										

Highest	8296	125895	129	194	15.2	9623	139138	42	34	14.5

Intermediate	11036	169469	238	238	15.4	11875	182635	73	79	15.4

Lowest	13119	220049	349	957	16.8	13922	245434	107	522	17.6

Total	32451	515413	716	1389	15.9	35420	567207	222	635	16.0

Empl. status										

Employed	25749	428906	483	804	16.7	25075	408378	124	196	16.3

Unemployed	1718	21244	90	62	12.4	1619	20153	19	18	12.4

Total	27467	450150	573	866	16.4	26694	428531	143	214	16.1

Income										

Highest	11121	180305	197	368	16.2	11439	185284	57	110	16.2

Intermediate	10656	171646	215	422	16.1	11853	190711	61	151	16.1

Lowest	10321	158719	281	533	15.4	11915	187955	100	355	15.8

Total	32098	510670	693	1323	15.9	35207	563950	218	616	16.0

The preliminary adjusted logistic regression analysis confirmed the associations between low socio-economic position and psychological distress for all indicators (see Additional file 2). The second preliminary analysis (see Additional file 3), based on the Cox proportional hazard model, demonstrated statistically significant hazard ratios for both unnatural and coronary heart disease mortality by psychological distress. Hazard ratios for psychological distress were higher for unnatural causes of death than for CHD mortality

### Contribution of psychological distress to educational differences in mortality

In the main Cox proportional hazard model analyses, we examined the contribution of the psychological distress variables to excess mortality by socio-economic position for each of the socio-economic variables separately (Tables 1-3). The hazard ratios for educational level in Table 1 present the effect of adjusting for psychological distress variables on the relative differences by educational level in unnatural and CHD mortality among men and women. In the base model for men, we found excess unnatural mortality in the intermediate and lowest educational levels, and excess CHD mortality in the lowest level of education. However, adjusting for measures of psychological distress, when considered both separately and simultaneously, resulted in a very modest reduction in the relative mortality difference by educational level (5-12%) in unnatural causes of death, and no change in CHD mortality (0-5%). In women, the level of education was statistically significantly associated only with CHD mortality, where the contribution of psychological distress variables was equivalent to men.

### Contribution of psychological distress to employment status differences in mortality

In the base model presented in Table 2, unemployment was associated with increased mortality in both genders. For unnatural cause of death, adjusting for psychological distress variables separately and simultaneously accounted for 11-31% of the excess mortality in unemployed men and 11-26% in women. Adjusting for all of the measures of psychological distress combined resulted in further reductions to the excess risk of unnatural mortality among the unemployed. Adjusting for psychological distress attenuated the association between employment status and CHD mortality at the most 9% among both men and women.

### Contribution of psychological distress to household income level differences in mortality

In the base model for mortality by household income level, we found a higher risk of mortality in the lowest income group compared to the highest income group among men and women in both unnatural and CHD mortality (Table 3). After controlling for the psychological distress variables separately or combined, psychological distress accounted for 4-16% of the differences in unnatural mortality among those at the lowest income level in both men and women. Again, the effect of adjustment for psychological distress measures in CHD mortality by income level appeared weak (0-5%).

## Discussion

Based on our results, we can conclude that psychological distress partly accounted for employment status and household income level differences in unnatural mortality (suicide, accidents and violence, and alcohol-related mortality) in both genders, and for educational level differences in unnatural mortality among men; among women no significant educational differences were found in unnatural mortality in the first place. The contribution of psychological distress variables to socio-economic differences in CHD mortality, on the other hand, was negligible.

The strength of our study is the nationally representative data from repeated population surveys, which was supplemented with extensive socio-economic register data and national causes of death register data, providing for a prospective study design with a 28-year follow-up. However, the cross-sectional measure of socio-economic factors and psychological distress variables allows for no conclusions about the direction of the association, that is, health selection versus causation, which may both contribute to the associations between socio-economic position and psychological factors [18].

The response rate of the survey is similar to that of other population surveys [48]. However, in our non-respondent analysis of this data [49] we found lower response rates for the lower educated. Total and cause specific (for example, alcohol, external causes, suicide) excess mortality rates were higher among survey non-respondents and this is partly explained by educational and income differences between respondents and non-respondents [50]. These results indicate that non-respondents have more severe illnesses, mental health problems and depression as well as unhealthy lifestyles, such as smoking and alcohol use. They also indicate that the comparability of the results of the different socio-economic groups may be biased and, therefore, the socio-economic differences may actually be stronger than those observed in this data. Additional analyses for respondents with missing data on psychological distress variables (N = 1129, 1,6%), although containing relatively small number, showed that those with missing data on psychological distress measures were also more likely to be in the lower SES groups.

One principal limitation of the study is that the measures of psychological distress are very simple self-reported single-item questions. These measures may cover a variety of transient or chronic psychological symptoms, a wide range of meanings from the temporary decrease of psychological well-being to deeply impaired, even life-threatening disorders. Therefore, the main focus of these indicators is not to detect clinical disorders but to reflect the subjective experience of mental health, and to study mental well-being at an extensive population level [43]. Nevertheless, single-item psychological distress variables demonstrated significant associations with cause-specific mortality, indicating that self-reported psychological distress have an implication for health. Another limitation concerning measures used in this study is the unemployed versus employed classification, which is a crude measure of employment status.

In the previous studies psychological factors only weakly or moderately mediate the relationship between SES and all-cause mortality [40,41]. In this study, we analysed three different measures of psychological distress and found some mediation for unnatural mortality and SES, and weak mediation for CHD mortality by employment status. It has been proposed that the excess CHD mortality among those in a lower socio-economic position is dependent on socio-economic differences in behavioural and biological risk factors, such as smoking, blood pressure and serum cholesterol levels [51]. A previous study based on the same data examined health behaviours as explanations for educational level differences in CHD mortality [47]. Health behaviours, most importantly smoking, physical activity and vegetable intake, explained about 50% of the educational differences in CHD mortality among men, but did not explain much of the differences among women. Compared to these results, psychological factors examined in the present study did not add to the contribution made by behavioural factors in explaining socio-economic differences in CHD mortality. However, psychological distress explaining some of the inequalities in suicide, accidents and violence, and alcohol-related mortality indicates that in these specific causes of death, poor mental health is related to more severe consequences in the lower socio-economic status groups than in the higher SES groups. It is possibly due to poor coping strategies of psychological distress in the lower SES. Obviously, that includes risky behaviour and, above all, heavy alcohol consumption which may be aimed at relieving psychological symptoms.

Theories and models which propose psychosocial factors as mediators in the SES-health relationship also emphasize that health status is the result of complex causes. Health behaviour, socio-demographic factors and early environmental, genetic, biomedical and medical factors are all seen as related to this phenomenon. Our results suggest that psychological distress may explain some of the cause-specific mortality disparities between socio-economic groups.

## Conclusions

Psychological distress partly accounted for socio-economic disparities in unnatural mortality, but notably less for CHD mortality. Improvement of psychological well-being in lower socio-economic groups may reduce some of the socio-economic disparities in cause-specific mortality. Especially, the possible mental health problems of the unemployed should be taken into account when searching for a means to decrease these inequalities. Further studies are needed to explicate the role and mechanisms of psychological distress in generating socio-economic differences, particularly in cause-specific mortality.

## Competing interests

The authors declare that they have no competing interests.

## Authors' contributions

KTM processed the data, carried out the statistical analyses and drafted the manuscript. TKML, TMH, AIO were involved in interpreting the data and drafting the manuscript. TPM supervised the first author and was involved in interpreting the data and drafting the manuscript. RSP was involved in data management, coordinated the study, supervised the first author and was involved in drafting the manuscript. All authors revised the text critically for important intellectual content and read and approved the final manuscript.

## Pre-publication history

The pre-publication history for this paper can be accessed here:

http://www.biomedcentral.com/1471-2458/11/138/prepub

## Supplementary Material

Additional file 1 **Appendix figure S1**. Conceptual framework of the study. Click here for file

Additional file 2 **Appendix table S1**. Logistic regression model (Odds Ratios, 95% Confidence Intervals) for psychological distress by socio-economic position. Males and females. Adjusted for age and study year. Click here for file

Additional file 3 **Appendix table S2**. Adjusted Cox proportional hazard model for the unnatural and CHD mortality in self-reported psychological distress. Adjusted for age, age squared and study year. Click here for file
