# Supplementary material for: The contribution of psychological distress to socio-economic differences in cause-specific mortality: a population-based follow-up of 28 years
Source: BMC Public Health. 2011 Feb 28;11:138. doi: 10.1186/1471-2458-11-138 (PMC3053248; doi:10.1186/1471-2458-11-138)
Supplement: Additional file 1 — Appendix figure S1. Conceptual framework of the study. [file 1471-2458-11-138-S1.PPT]

## Slide 1
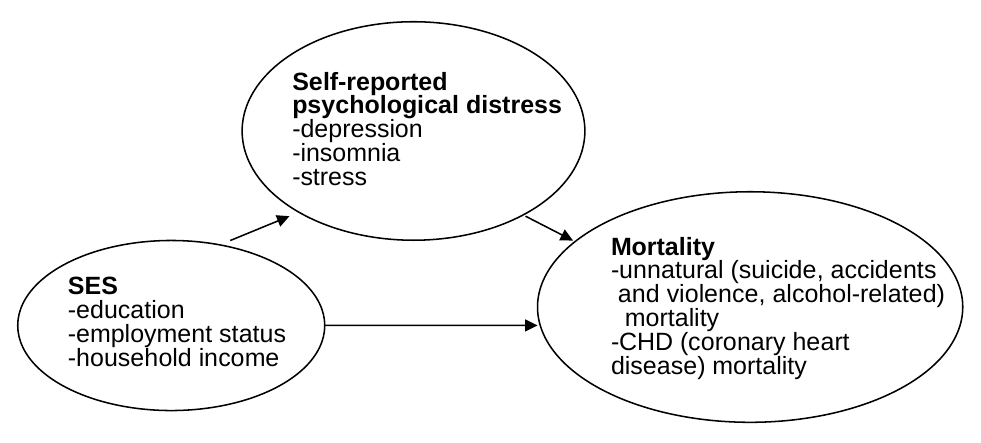

Self-reported
psychological distress
-depression
-insomnia
-stress
SES
-education
-employment status
-household income
Mortality
-unnatural (suicide, accidents and violence, alcohol-related) mortality
-CHD (coronary heart
disease) mortality
