# Supplementary material for: The contribution of psychological distress to socio-economic differences in cause-specific mortality: a population-based follow-up of 28 years
Source: BMC Public Health. 2011 Feb 28;11:138. doi: 10.1186/1471-2458-11-138 (PMC3053248; doi:10.1186/1471-2458-11-138)
Supplement: Additional file 2 — Appendix table S1. Logistic regression model (Odds Ratios, 95% Confidence Intervals) for psychological distress by socio-economic position. Males and females. Adjusted for age and study year. [file 1471-2458-11-138-S2.DOC]

| **Appendix table 1 - Logistic regression model (Odds Ratios, 95% Confidence Intervals) for psychological distress by socio-economic**  **position. Males and females.** Adjusted for age and study year. | | | | | | | | | | | | | | | | | | |
| --- | --- | --- | --- | --- | --- | --- | --- | --- | --- | --- | --- | --- | --- | --- | --- | --- | --- | --- |
| Variable | Depression | | |  | |  | Insomnia | | |  | |  | Stress | |  |  |  |  |
|  | Males | |  | Females | |  | Males | |  | Females | |  | Males | |  | Females | |  |
|  | OR | CI | 95% | OR | CI | 95% | OR | CI | 95% | OR | CI | 95% | OR | CI | 95% | OR | CI | 95% |
| Education |  |  |  |  |  |  |  |  |  |  |  |  |  |  |  |  |  |  |
| Highest | 1.00 |  |  | 1.00 |  |  | 1.00 |  |  | 1.00 |  |  | 1.00 |  |  | 1.00 |  |  |
| Intermediate | 1.20 | 1.10 | 1.30 | 1.08 | 1.01 | 1.16 | 1.11 | 1.03 | 1.20 | 0.99 | 0.92 | 1.07 | 1.26 | 1.04 | 1.52 | 1.22 | 1.01 | 1.47 |
| Lowest | 1.26 | 1.16 | 1.37 | 1.19 | 1.10 | 1.28 | 1.17 | 1.08 | 1.26 | 1.08 | 1.01 | 1.17 | 1.37 | 1.13 | 1.65 | 1.47 | 1.21 | 1.78 |
| Employment status |  |  |  |  |  |  |  |  |  |  |  |  |  |  |  |  |  |  |
| Employed | 1.00 |  |  | 1.00 |  |  | 1.00 |  |  | 1.00 |  |  | 1.00 |  |  | 1.00 |  |  |
| Unemployed | 3.17 | 2.83 | 3.54 | 2.31 | 2.06 | 2.59 | 2.31 | 2.07 | 2.57 | 1.78 | 1.58 | 2.00 | 4.10 | 3.35 | 5.02 | 2.77 | 2.18 | 3.51 |
| Income |  |  |  |  |  |  |  |  |  |  |  |  |  |  |  |  |  |  |
| Highest | 1.00 |  |  | 1.00 |  |  | 1.00 |  |  | 1.00 |  |  | 1.00 |  |  | 1.00 |  |  |
| Intermediate | 1.25 | 1.15 | 1.35 | 1.05 | 0.98 | 1.13 | 0.98 | 0.91 | 1.05 | 0.94 | 0.88 | 1.00 | 1.15 | 0.95 | 1.39 | 1.15 | 0.95 | 1.39 |
| Lowest | 1.60 | 1.48 | 1.73 | 1.35 | 1.27 | 1.45 | 1.24 | 1.15 | 1.32 | 1.13 | 1.06 | 1.21 | 2.00 | 1.68 | 2.34 | 2.10 | 1.76 | 2.49 |
