# Supplementary material for: The contribution of psychological distress to socio-economic differences in cause-specific mortality: a population-based follow-up of 28 years
Source: BMC Public Health. 2011 Feb 28;11:138. doi: 10.1186/1471-2458-11-138 (PMC3053248; doi:10.1186/1471-2458-11-138)
Supplement: Additional file 3 — Appendix table S2. Adjusted Cox proportional hazard model for the unnatural and CHD mortality in self-reported psychological distress. Adjusted for age, age squared and study year. [file 1471-2458-11-138-S3.DOC]

| **Appendix table 2 - Adjusted Cox proportional hazard model for the unnatural and CHD mortality in self-reported psychological distress.** Adjusted for age, age squared and study year | | |
| --- | --- | --- |
|  | Unnatural causes of mortality | CHD mortality |
|  | Hazard Ratio  (95% CI) | Hazard Ratio  (95% CI) |
| Men | N=716 | N=1389 |
| No depression | 1.00 | 1.00 |
| Depression | 2.36 (2.00-2.79) | 1.35 (1.17-1.56) |
| No stress | 1.00 | 1.00 |
| Stress | 3.37 (2.54-4.49) | 1.76 (1.32-2.35) |
| No insomnia | 1.00 | 1.00 |
| Insomnia | 2.93 (2.51-3.41) | 1.33 (1.17-1.51) |
| Women | N=222 | N=634 |
| No depression | 1.00 | 1.00 |
| Depression | 2.43 (1.85-3.21) | 1.43 (1.19-1.72) |
| No stress | 1.00 | 1.00 |
| Stress | 4.04 (2.49-6.55) | 2.34 (1.59-3.44) |
| No insomnia | 1.00 | 1.00 |
| Insomnia | 2.29 (1.73-3.04) | 1.25 (1.06-1.48) |
